# Effect of arthrocentesis on the clinical outcome of various treatment methods for temporomandibular joint disorders

**DOI:** 10.1186/s40902-019-0227-z

**Published:** 2019-10-22

**Authors:** Chang-Woo Kim, Sung-Jae Lee, Euy-Hyun Kim, Dong-Keon Lee, Mong-Hun Kang, In-Seok Song, Sang-Ho Jun

**Affiliations:** Department of Oral and Maxillofacial Surgery, Korea University Anam Hospital, 73, Inchon-ro, Seongbuk-gu, Seoul, 02841 Republic of Korea

**Keywords:** Temporomandibular joint arthrocentesis, Temporomandibular disorders

## Abstract

**Background:**

We evaluated the improvement of pain and the increase in mouth opening after temporomandibular joint arthrocentesis and the possible association with various factors such as previous splint treatment, medication, and diagnosis.

**Results:**

We studied 57 temporomandibular joint disorder patients who underwent arthrocentesis at Korea University Anam Hospital. These patients (24 males and 33 females, aged between 15 and 76 years) underwent arthrocentesis that was performed by one surgeon. The degree of mouth opening (assessed using the maximum mouth opening: MMO) and pain (assessed using the visual analog scale: VAS) were assessed pre- and post-arthrocentesis. The study also investigated whether treatment modalities other than arthrocentesis (medication and appliance therapy) were performed. Statistical analysis revealed that there was a significant difference in mouth opening and pain after temporomandibular joint arthrocentesis. Preoperative appliance therapy affected the results of arthrocentesis, but it was not statistically significant. With regard to pain relief, preoperative diagnosis did not show a significant difference. However, with regard to maximum mouth opening, patients with disc displacement without reduction with limited mouth opening (closed lock) showed the highest recovery (11.13 mm).

**Conclusion:**

The average of MMO increase after arthrocentesis was 9.10 mm, and patients with disc displacement without reduction with locking (closed lock) showed most recovery in maximum mouth opening and it was statistically significant. The average pain relief of patients after arthrocentesis was 3.03 in the VAS scale, and patients using anterior repositioning splint (ARS) preoperatively showed the most pain relief.

## Background

Temporomandibular joint (TMJ) disorder (TMD) is a term used to define disorders occurring in the masticatory muscles, TMJ, and surrounding tissues [1]. Regardless of the tissue affected, TMDs show similar signs and symptoms such as pain around the ear and masticatory muscles [2]. The initial stage of TMD is characterized by normal maximum mouth opening with joint sounds, which gradually decreases with limitation of mouth opening. The advanced stage of TMD is characterized by dislocation of the articular disc and restricted sliding movements [3].

Patients with intra-articular TMDs present with various symptoms. An imbalance in the physiological relationship between the articular disc and condylar head causes various symptoms [4]. Intra-articular TMDs are classified into six types according to the Research Diagnostic Criteria for Temporomandibular Disorders (RDC/TMD): “disc displacement with reduction”; “disc displacement with reduction with intermittent locking”; “disc displacement without reduction with limited opening”; “disc displacement without reduction, without limited opening”; “degenerative joint disease”; and “subluxation” [5]. Al-Khotani et al. [6] show that disc displacement with reduction is the most common TMJ problem.

TMJ arthrocentesis and lavage originated from the successful results of TMJ arthroscopy. Since the first publication on arthrocentesis by Nitzan and Dolwick in 1991 [7], TMJ arthrocentesis received widespread acceptance, as a minimally invasive surgical procedure for TMDs refractory to other conservative treatments. In order to increase mouth opening and reduce pain, arthrocentesis was performed by inserting two catheters into the upper joint space under local anesthesia and irrigation with saline. This treatment proved to be very effective for disc displacement without reduction with limited mouth opening (closed lock) [8]. Compared with other surgical procedures, TMJ arthrocentesis has been reported to be effective in reducing pain and increase in mouth opening with low incidence of complications [9]. Nitzan et al. reported that patients showed a significant increase in mouth opening (*P* < 0.001). Before the procedure, they showed a mean mouth opening of 24.1 ± 5.6 mm that increased to 42.7 ± 4 mm after arthrocentesis. The mean preoperative VAS (visual analog scale) score was 8.75 ± 2.82, within the scale of 0 to 15, and it decreased significantly (*P* < 0.001) to 2.31 ± 2.55 after the procedure [8].

Appliance therapy was revealed effective in TMD patients with arthrogenous pain, with the purpose of reducing inflammation and joint load [10]. Zhang et al. reported in their systematic review that splint therapy can increase the MMO and reduce the frequency of pain in TMD patients [11]. Centric relation splint (CRS) and anterior repositioning splints (ARS) are the most common splint used in TMD patients. CRS is the most widely accepted nonsurgical treatment of TMDs which can improve many of the clinical symptoms of TMDs [12]. ARS can be used when the symptoms persist after the CRS application. Many articles have reported the efficacy of ARS for increase mouth opening in the disc displacement patients [4, 13, 14]. However, there is only scarce study about the relationship between splint therapy and arthrocentesis results and they only studied about one type of splint [15, 16].

A muscle relaxant affects skeletal muscle function and decreases muscle tones. The decreased muscle tone can prevent the unnecessary forces that affect the TMJ, thus reducing the pain and giving stability to surrounding tissues. The muscle relaxants are believed to action by treating either spasticity secondary to upper motor neuron syndromes or muscular pain and spasms secondary to peripheral musculoskeletal conditions [17]. There are only few articles studied about the relationship between muscle relaxant and the outcome of TMJ arthrocentesis.

In this article, we evaluated the relationship between the decrease in pain and increase in mouth opening after TMJ arthrocentesis and the various factors such as previous splint treatment, medication, and diagnosis.

## Materials and methods

### Population

This study was conducted on patients who visited Korea University Anam Hospital between January 2016 and June 2018 and were diagnosed with degenerative joint disease and disc displacement with and without reduction on radiographic and clinical evaluation. TMJ arthrocentesis was performed by one surgeon, and mouth opening and the degree of pain before and after the operation were examined. We also investigated whether other treatments (e.g., device therapy) were performed before arthrocentesis. This study was approved by Korea University Anam Hospital in Seoul, South Korea: IRB number 2018AN0427, and informed consent was exempted because this study was a retrospective study.

### Inclusion and exclusion criteria

The sample was composed of individuals of both genders older than 15 years. Patients with signs and symptoms of intra-articular TMDs and degenerative joint disease and refractory to conservative treatment were included. They were diagnosed by the Research Diagnostic Criteria for Temporomandibular Disorders (RDC/TMD). Patients with previous surgery, hypoplasia and/or malignant neoplasm of the mandibular condyle, bone ankylosis, drug allergy, history of psychosomatic illness, or pregnant and lactating patients were excluded.

### Procedure

The mouth opening and degree of pain were measured before TMJ lavage. The first step of TMJ arthrocentesis was extensive disinfection of the surgical site; a line was then drawn connecting the middle portion of the tragus and the outer canthus of the eye.

Articular fossa and eminence were marked at 10 mm and 20 mm respectively, in front of the tragus, along the canthal-tragus line, and vertically bellow at 2 mm and 10 mm respectively. Through palpation of the two reference points, the condyle and articular eminence were marked with methylene blue solution.

To reduce pain when inserting the needle, subcutaneous infiltration anesthesia was performed with 0.5 ml of 2% lidocaine using a dental anesthesia syringe.

After opening the mouth of the patient, a 2-mL saline syringe was inserted with a 26G needle into the upper joint space, and saline was inserted with a gentle force. When 2 mL saline was aspirated again, it indicates that the needle is inserted into the proper place. The second needle was inserted in the same way in the same direction. After the needle was inserted, irrigation was performed for 30 min with 500 mL saline. At this time, the fluid was located at 1.5 m above the patient who is in supine position.

Immediately after surgery, the surgeon injected hyaluronic acid into the operative site of the patient, and the distance between the maxillary and mandibular central incisors was measured to determine the MMO. Pain was recorded using the VAS, which is a subjective assessment. Postoperative MMO and VAS was measured 4 days after the procedure when patients came to the clinic for follow-up. The splint therapy was continued after 4-day follow-up.

### Splint therapy

CRS and ARS were used to some patients in this study. CRS was used to reduce the bite force and joint load. ARS was used in patients with disc displacement, patients whose symptom persists after physical therapy or joint inflammation, and patients with severe inflammation to reduce the occlusal load. Splints were used prior to arthrocentesis. The period and duration of the appliance use varied depending on each patient’s symptom. Patients with severe symptoms wore the splint for 24 h (less than 1 week), and when the symptoms subsided, they were told to wear the splint during nighttime only.

### Muscle relaxant

Muscle relaxant was prescribed to patients with muscle tenderness or pain. Thiosina Tab. (thiocolchicoside 4 mg, aescin 20 mg) was used to patients three times a day. The medicine was prescribed more than 7 days depending on the symptom of the patients. Muscle relaxant was prescribed with painkiller (Somalgen Tab, talniflumate 370 mg) for the same days.

### Statistical analysis

SPSS for Windows**®** (version 12.0, SPSS Inc., Chicago, IL, USA) was used for statistical analysis. To evaluate the result of arthrocentesis, paired *t* test was conducted. To compare the result of arthrocentesis categorized by various factors, *t* test and ANOVA were used. *P* < 0.05 was considered statistically significant in this study.

## Results

The 57 patients with TMD met the eligibility criteria and underwent arthrocentesis (24 male patients and 33 female patients). Of the 57 cases with TMJ lavage, 6 cases were performed on both sides and 51 cases were unilateral; 27 cases were of the right side and 24 were left.

The average age of the patients was 38.65 ± 17.59 years old (Table 1). As a result of arthrocentesis, the preoperative pain score of 4.77 ± 1.98 decreased to 1.74 ± 1.89, and this difference was statistically significant (*P* < 0.05) (Table 2). The MMO increased from 37.25 ±7.40 to 46.35 ± 7.39 after arthrocentesis, and this change was statistically significant (*P* < 0.05) (Table 2). Patients were sorted into two groups based on whether they underwent appliance therapy before arthrocentesis. Twenty-five out of 57 patients underwent splint therapy before arthrocentesis. Patients who used TMJ splint before arthrocentesis showed slightly better improvement in MMO (9.76) and a better decrease in VAS score (3.42) than the other group (8.59, 2.73), but this change was not statistically significant (Tables 3 and 4).

**Table 1:** Baseline characteristics

	Patients (*N* = 57)
Age (year)	
*N*	57
Mean (SD)	38.65 (17.59)
Median (Q1, Q3)	32.00 (23.00, 54.00)
(Min, max)	(15.00, 76.00)
Sex, *n* (%)	
Male	24 (42.11)
Female	33 (57.89)

**Table 2:** Result of arthrocentesis

	*N*	Pre		Post		*P* value*
		Mean	(SD)	Mean	(SD)	
VAS	57	4.77	(1.98)	1.74	(1.89)	< .0001
MMO	57	37.25	(7.40)	46.35	(7.39)	< .0001

*P* < 0.05 was considered statistically significant

*Pre* preoperative, *post* postoperative, *VAS* visual analog scaled, *MMO* maximum mouth opening

* *p*-value calculated by paired t-test

**Table 3:** Improvement of VAS score due to various factor

	VAS								
	*N*	Pre		Post		*P* value*	Difference^†^ of VAS		*P* value
		Mean	(SD)	Mean	(SD)		Mean	(SD)	
Appliance treatment									0.1768^a^
No splint	32	4.48	(1.94)	1.75	(1.92)	< .0001	− 2.73	(1.88)	
Splint tx	25	5.14	(2.02)	1.72	(1.88)	< .0001	− 3.42	(1.87)	
Type of appliance tx									0.4002^b^
No splint	32	4.48	(1.94)	1.75	(1.92)	< .0001	− 2.73	(1.88)	
ARS	9	5.00	(2.87)	1.33	(2.18)	0.0023	− 3.67	(2.50)	
ARS and CRS	15	5.23	(1.50)	2.07	(1.71)	< .0001	− 3.17	(1.46)	
Dx									0.9568^b^
DD with locking	32	4.81	(2.30)	1.84	(1.92)	< .0001	− 2.97	(1.84)	
DD without locking	15	4.80	(1.74)	1.67	(1.72)	< .0001	− 3.13	(2.07)	
DJD	10	4.60	(1.26)	1.50	(2.17)	0.0008	− 3.10	(1.97)	
Muscle relaxants									0.6760^a^
No	11	5.00	(2.41)	2.18	(1.78)	0.0004	− 2.82	(1.78)	
Yes	46	4.72	(1.90)	1.63	(1.91)	< .0001	− 3.09	(1.93)	
Locking period									0.0697^a^
< 6 months	34	4.46	(2.24)	1.79	(1.97)	< .0001	− 2.66	(1.96)	
≥ 6 months	23	5.24	(1.44)	1.65	(1.80)	< .0001	− 3.59	(1.68)	

*P* < 0.05 considered statistically significant

*DD* disc displacement, *DJD* degenerative joint disease, *ARS* anterior repositioning splint, *CRS* centric relation splint

*P value calculated by paired *t* test

^†^Difference = post − pre

^a^*P* value calculated by t-test

^b^*P* value calculated by ANOVA

**Table 4:** Improvement in MMO due to various factor

	MMO								
	*N*	Pre		Post		*P* value*	Difference^†^ of MMO		*P* value
		Mean	(SD)	Mean	(SD)		Mean	(SD)	
Appliance treatment									0.4792^a^
No splint	32	36.84	(8.33)	45.44	(8.64)	< .0001	8.59	(6.61)	
Splint tx	25	37.76	(6.12)	47.52	(5.33)	< .0001	9.76	(5.46)	
Type of appliance tx									0.5993^b^
No splint	32	36.84	(8.33)	45.44	(8.64)	< .0001	8.59	(6.61)	
ARS	9	38.11	(5.58)	46.89	(5.80)	0.0007	8.78	(4.94)	
ARS and CRS	15	37.27	(6.69)	47.80	(5.39)	< .0001	10.53	(5.94)	
Dx									0.0156^b^
DD with locking	32	33.38	(6.97)	44.50	(8.14)	< .0001	11.13	(6.73)	
DD without locking	15	42.40	(4.40)	48.80	(5.56)	< .0001	6.40	(4.27)	
DJD	10	41.90	(4.61)	48.60	(5.99)	0.0003	6.70	(3.80)	
Muscle relaxants									0.6190^a^
No	11	38.09	(6.47)	46.36	(5.18)	0.0005	8.27	(5.37)	
Yes	46	37.04	(7.65)	46.35	(7.87)	< .0001	9.30	(6.31)	
Locking period									0.6441^a^
< 6 months	34	37.26	(8.06)	46.06	(8.37)	0.0005	8.79	(5.80)	
≥ 6 months	23	37.22	(6.46)	46.78	(5.78)	< .0001	9.57	(6.64)	

*P* < 0.05 was considered statistically significant

*DD* disc displacement, *DJD* degenerative joint disease, *ARS* anterior repositioning splint, *CRS* centric relation splint

*p value calculated by paired t-test

^†^Difference = post − pre

^a^*P* value calculated by *t* test

^b^*P* value calculated by ANOVA

Patients treated with ARS showed most pain relief (3.67 ± 2.50) after arthrocentesis (Table 3). However, there was no statistical significance (*P* = 0.400). With regard to MMO, the patients who were treated by both ARS and CRS showed more improvement (10.53 ± 5.94) than other groups (treated without appliance and treated with ARS) (Table 4). However, it was not statistically significant (*P* = 0.599). One patient was excluded in statistics because only one patient was treated with CRS.

Patients were diagnosed by the RDC/TMD criteria. Patients were divided into the following three groups: disc displacement with locking group (RDC/TMD criteria; disc displacement without reduction with locking-closed lock), disc displacement without locking group (RDC/TMD criteria; disc displacement with reduction, disc displacement with reduction with intermittent locking, disc displacement without reduction without locking), and degenerative joint disease group. There was no statistically significant association between preoperative diagnosis and pain relief (Table 3). However, patients with disc displacement without reduction with limited mouth opening (closed lock) showed the highest improvement in mouth opening (11.13 ± 6.73) and it was significantly higher (*P* < 0.05) than those with degenerative joint disease and those with disc displacement without limited mouth opening (Table 4).

Forty-six out of 57 patients took a muscle relaxant to reduce the TMJ symptoms before arthrocentesis (Tables 3 and 4). These patients showed slightly better improvements in MMO and VAS scores, but it was not significant (Tables 3 and 4).

Patients were also classified by the locking period. Locking was defined as subjective mouth opening limitation that patients say. To evaluate the efficacy of arthrocentesis on acute and chronic locking state, patients were divided into two groups depending on the locking period. Twenty-three out of 57 patients suffered from TMJ locking for more than 6 months (Tables 3 and 4). Patients with TMJ locking more than 6 months showed better results after arthrocentesis. However, this was not statistically significant when compared with the patients with TMJ locking period less than 6 months.

## Discussion

TMDs originate from the joint itself or arise due to any pathology of the muscles [18]. The TMJ can adjust to the function of the jaws continuously by remodeling [19]. However, when the functional load exceeds the regenerative capacity of the joints, the remodeling becomes insufficient and results in structural change. These changes cause deformities in the TMJ, resulting in clinical symptoms.

Arthrocentesis has been demonstrated to be effective in treating disc displacement without reduction [20]. Arthrocentesis and lavage have been suggested to be useful for the management of other TMDs. Murakami et al. [21] reported that arthrocentesis showed favorable results in mitigating the symptoms of the advanced stage of internal derangement. It should be pointed out that arthrocentesis is effective in treating symptomatic TMDs [9]. Patients with intra-articular TMDs were divided into three groups in this study. Patients with disc displacement were sorted by whether they have limited mouth opening (MMO < 40) or not. Patients with degenerative joint disease (DJD) were the third group. Late-stage DJD with acquired mandibular retrognathia requires surgical management; however, early-stage DJD without significant bony changes should be treated with nonsurgical methods or minimally invasive surgical methods, such as arthrocentesis and arthroscopy [22]. In this study, arthrocentesis was effective in the early stage of DJD patient in improving range of MMO and reducing pain just as the formal study by Nitzan et al. [9]. When comparing the result of arthrocentesis in the three groups, the disc displacement with locking group showed significant MMO recovery than the other two groups (Table 4). The mechanism of MMO recovery in closed lock patients is uncertain, because the cause of the limited motion and pathology is still unknown [23]. The relieve of negative pressure on the disc, adhesion, and surface friction might be the reason for the significant MMO recovery of the closed lock group.

TMJ splints such as ARS and CRS are most widely used conservative treatment that relieve the joint and muscle pain based on TMD education [14]. It was demonstrated that stabilization splints reduce the number of painful muscles and the degree of the pain after short use [24]. ARS was used for patients with anterior disc displacement and patients with onset of limited ability to mouth opening. To reduce the occlusal instability, ARS was used for only a short time and the use of stabilization splint was followed. CRS was used for patients with muscle hyperactivity and parafunctional activity in this study. There was no significant additional pain relief and MMO recovery by applying the occlusal splint before arthrocentesis, as shown in Tables 3 and 4. The result does not concur with the formal study that concluded arthrocentesis is more effective when used in conjunction with splint therapy [18, 25]. In this study, patients underwent appliance treatment in various periods and duration. Also, only short-term results were analyzed. In a long-term study by Lee et al, simultaneous wearing of splint after arthrocentesis showed a better result than preoperative splint treatment [16]. So further long-term study is needed for determining the relationship between the splint use and arthrocentesis.

There were studies about arthrocentesis that prescribed muscle relaxant after the procedure [26, 27]. However, there were scarce studies comparing patients with or without muscle relaxant. The results of arthrocentesis with or without muscle relaxant were statistically analyzed in our study. There was no statistical difference between the two groups in short-term evaluation.

Locking period was one of the factors that were analyzed in this study. The result was that patient with locking more than 6 months showed slightly better results, but it was not statistically significant (Tables 3 and 4). However, the study was conducted in a short period to evaluate the relationship between the efficacy of arthrocentesis and locking period. Thus, further study is needed to evaluate the relationship between locking period and TMJ arthrocentesis.

We investigated the MMO to evaluate the function of the TMJ. However, this study lacks information about the lateral movements of the jaws and TMJ. Hence, further study is needed to evaluate the improvement in TMJ function after arthrocentesis. The study was conducted to short-term follow-up, so it could not evaluate the long-term results.

In this study, there were no complications such as facial nerve injury, pre-auricular hematoma, superficial temporal artery injury, and needle breakage in the joint [28] after TMJ arthrocentesis.

## Conclusion

The average MMO increase after arthrocentesis was 9.10 mm, and patients with disc displacement without reduction with locking (closed lock) showed most recovery in MMO and showed statistically significant results. Among other factors, patients who used ARS and CRS preoperatively showed the second-best results in MMO.

The average pain relief of patients after arthrocentesis was 3.03 in VAS scale, and patients using ARS preoperatively showed the most pain relief.

## Data Availability

Please contact the author for data requests.

## Abbreviations

MMO: Maximum mouth opening
TMDs: Temporomandibular disorders
TMJ: Temporomandibular joint
VAS: Visual analog scale
